# Multi-patient study for coronary vulnerable plaque model comparisons: 2D/3D and fluid–structure interaction simulations

**DOI:** 10.1007/s10237-021-01450-8

**Published:** 2021-03-23

**Authors:** Qingyu Wang, Dalin Tang, Liang Wang, Akiko Meahara, David Molony, Habib Samady, Jie Zheng, Gary S. Mintz, Gregg W. Stone, Don P. Giddens

**Affiliations:** 1 School of Biological Science and Medical Engineering, Southeast University, Nanjing, 210096 China; 2 Mathematical Sciences Department, Worcester Polytechnic Institute, 100 Institute Road, Worcester, MA 01609 USA; 3 The Cardiovascular Research Foundation, Columbia University, New York, NY 10022 USA; 4 Department of Medicine, Emory University School of Medicine, Atlanta, GA 30307 USA; 5 Mallinckrodt Institute of Radiology, Washington University, St. Louis, MO 63110 USA; 6 Icahn School of Medicine At Mount Sinai, The Zena and Michael A. Wiener Cardiovascular Institute, New York, NY 10019 USA; 7 Department of Biomedical Engineering, The Wallace H. Coulter, Georgia Institute of Technology, Atlanta, GA 30332 USA

**Keywords:** Vulnerable plaque, VH-IVUS, Patient-specific model, Models comparison

## Abstract

Several image-based computational models have been used to perform mechanical analysis for atherosclerotic plaque progression and vulnerability investigations. However, differences of computational predictions from those models have not been quantified at multi-patient level. In vivo intravascular ultrasound (IVUS) coronary plaque data were acquired from seven patients. Seven 2D/3D models with/without circumferential shrink, cyclic bending and fluid–structure interactions (FSI) were constructed for the seven patients to perform model comparisons and quantify impact of 2D simplification, circumferential shrink, FSI and cyclic bending plaque wall stress/strain (PWS/PWSn) and flow shear stress (FSS) calculations. PWS/PWSn and FSS averages from seven patients (388 slices for 2D and 3D thin-layer models) were used for comparison. Compared to 2D models with shrink process, 2D models without shrink process overestimated PWS by 17.26%. PWS change at location with greatest curvature change from 3D FSI models with/without cyclic bending varied from 15.07% to 49.52% for the seven patients (average = 30.13%). Mean Max-FSS, Min-FSS and Ave-FSS from the flow-only models under maximum pressure condition were 4.02%, 11.29% and 5.45% higher than those from full FSI models with cycle bending, respectively. Mean PWS and PWSn differences between FSI and structure-only models were only 4.38% and 1.78%. Model differences had noticeable patient variations. FSI and flow-only model differences were greater for minimum FSS predictions, notable since low FSS is known to be related to plaque progression. Structure-only models could provide PWS/PWSn calculations as good approximations to FSI models for simplicity and time savings in calculation.

## Introduction

Computational models are powerful tools that have been used to perform mechanical analysis on atherosclerotic plaques and identify risk factors that may be related to plaque progression and rupture (Cardoso et al. 2014; Friedman et al. 2010; Gijsen et al. 2014; Holzapfel et al. 2014; Tang et al. 2014). Results from different models are influenced by many factors including plaque morphology and components, material properties, and modeling assumptions (Tang et al. 2014). With advances of medical imaging technologies, it is possible to obtain plaque morphology in vivo (Mintz et al. 2001; Nair et al. 2002). Two-dimensional (2D) image-based plaque solid models have previously been used to calculate stress/strain conditions of atherosclerotic plaques and to study their relationship to plaque progression and rupture (Li et al. 2006; Loree et al. 1992; Richardson et al. 1989). Considering the three-dimensional (3D) geometric structure of plaque, 3D structure-only models, 3D fluid-only models and 3D fluid–structure interaction (FSI) model have been used for the mechanical analysis of 3D plaque structure (Bluestein et al. 2008; Holzapfel et al. 2002; Ohayon et al. 2005; Samady et al. 2011; Stone et al. 2012; Tang et al. 2004; Teng et al. 2010). However, because of the complex geometry and composition of plaque, the construction of 3D plaque models is time-consuming. Various modeling strategies, including 2D structure-only model, 3D structure-only model and 3D FSI model, have been compared to investigate the differences in stress and strain analysis (Huang et al. 2014; Tang et al. 2004; Wang H et al. 2015). To improve the 2D model, a 3D thin-layer (TL) modeling method was used to replace 3D FSI model (Guo et al. 2018; Huang et al. 2016). However, previously published comparison of models mostly analyzed a single patient or models with idealized geometries. Multi-patient studies are more likely to demonstrate differences between models and according to patient variations.

For models based on in vivo data, it is important to obtain the initial zero-stress geometry of the plaque (Delfino et al. 1997; Holzapel et al. 2007; Huang et al. 2009; Ohayon et al. 2007; Pierce et al. 2015; Speelman et al. 2009; Wang L et al. 2017). Obtaining vessel’s initial zero-stress state from its in vivo stressed geometric state requires quantifying the artery opening angle, axial and circumferential shrinkages (Wang L. et al. 2017). Circumferential shrinkage is defined as the circumferential (radial) contraction ratio that causes the vessel to change from in vivo geometry to no-load geometry in circumferential direction. Circumferential shrinkage may be quantified from vessel deformation under pulsating pressure conditions. A shrink–stretch process was proposed to obtain vessel no-load morphology (ignoring opening angle) from its in vivo image data (Huang et al. 2009). Quantifying artery opening angle and axial stretch ratio for in vivo data is not possible due to absent tissue samples. Existing ex vivo data from available literature are often used for model constructions as an acceptable simplification.

For coronary plaques, ventricle contraction and motion cause vessel curvature change which has considerable impact on plaque biomechanical conditions. Yang et al. introduced 3D FSI model with cyclic bending to include cardiac motion for mechanical analysis of coronary plaques (Yang et al. 2009). Due to patient variations, it is important to perform multi-patient studies to evaluate the effect of cyclic bending on plaque mechanical behaviors.

In the present study, in vivo VH-IVUS images and X-ray angiographic data of coronary plaques from seven patients were obtained for model construction. Seven different models were constructed for all seven patients for comparisons. Our goals were to quantify: (1) the influence of circumferential shrink process on 2D model calculation in different patients; (2) the impact of cyclic bending process on 3D FSI model in different patients; (3) the differences between 2D model, 3D TL model and 3D FSI model in different patients; (4) the differences between 3D structure-only model and 3D FSI model in different patients; (5) the differences between 3D fluid-only model and 3D FSI model in different patients. Patient variations for those model differences were also quantified.

## Data, models and methods

### In vivo IVUS data acquisition

**Fig. 1:**
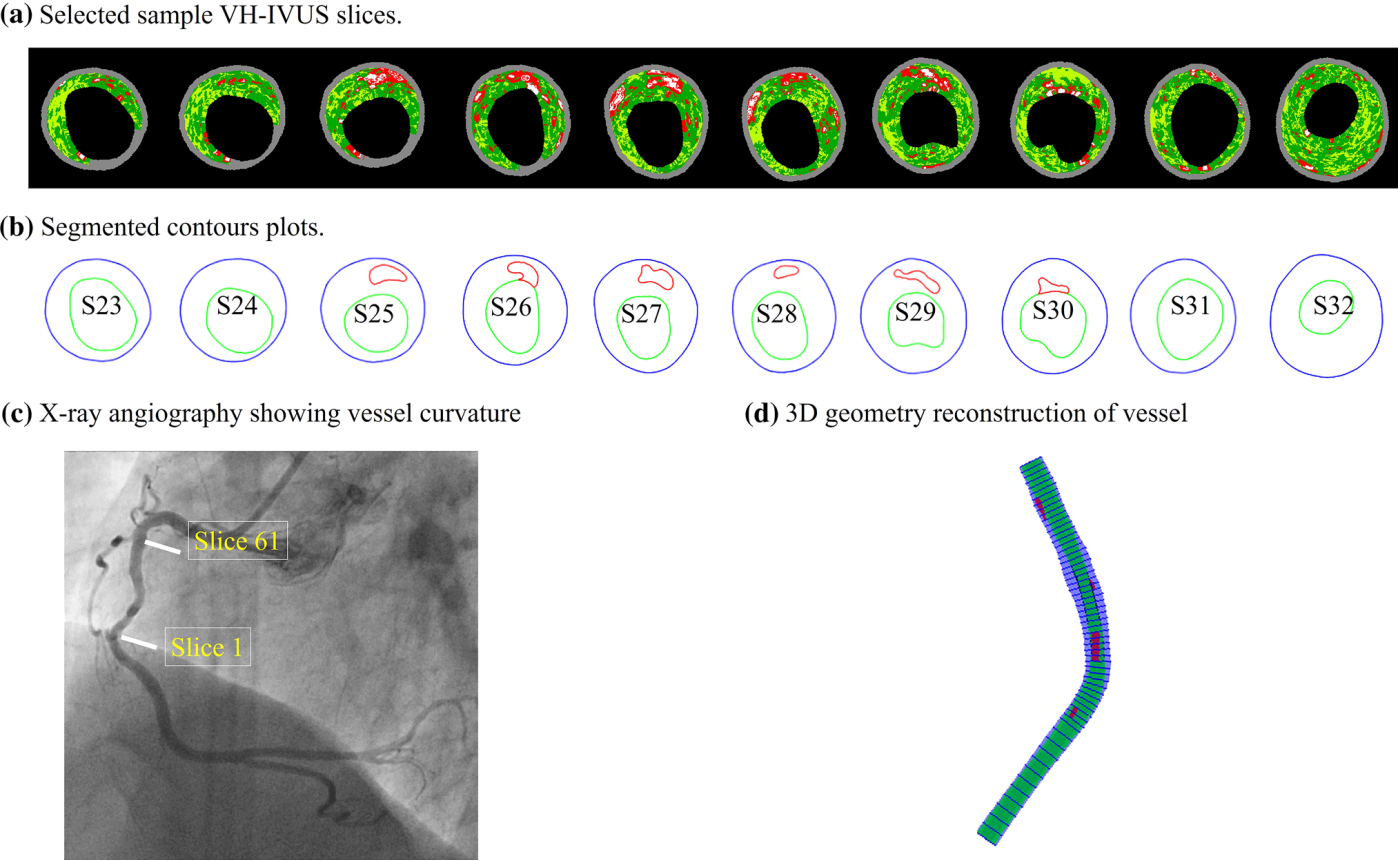
Selected sample VH-IVUS slices, segmented contour plots, X-ray angiographic image from a patient and the 3D vessel geometry reconstruction. Colors in VH-IVUS images: red, lipid; white, calcification; dark green, fibrous; light green, fibro-fatty

IVUS with radiofrequency “virtual” histology (VH-IVUS) data of coronary plaque data were acquired from seven patients (mean age: 62, 6 males) using a synthetic-aperture-array, 20-MHz, 3.2-French catheter (Eagle Eye, Volcano Corporation, Rancho Cordova, California) at the Cardiovascular Research Foundation (CRF) with informed consent obtained. Morphological information of seven patients is shown in Table 1. Locations of the coronary artery stenosis and vessel curvature were obtained from X-ray angiogram data. The centerline of each frame of X-ray angiography film was extracted to describe the vessel curvature. The maximum vessel curvature and the minimum vessel curvature were selected from a cardiac cycle to simulate the cardiac cyclic bending of the coronary. Figure 1 shows selected VH-IVUS images, their segmented contours, the X-ray angiography image, and the re-constructed 3D geometry of the vessel.

**Table 1:** Patient information

Patient	Age	Gender	BP (mmHg)	L-vessel (mm)	Number of slices	Stenosis (%)	PB (%)
P1	71.3	Male	70–125	70.58	59	45.97	73.52
P2	72.3	Male	70–125	70.00	61	61.87	82.51
P3	67.5	Male	70–120	28.96	56	5.79	58.23
P4	51.6	Male	60–135	33.92	64	38.73	68.45
P5	51	Male	97–144	35.37	40	35.59	69.08
P6	67	Female	70–110	55.02	44	56.57	78.10
P7	52.1	Male	60–135	33.98	64	43.26	73.61

L-Vessel: vessel segment length

Stenosis = (1 − (min lumen area/inlet lumen area)) $\times \hspace{0.17em}$100%

*PB* Plaque burden = [(wall area—lumen area)/wall area] $\times \hspace{0.17em}$100%

### List of models, governing equations, boundary conditions and material models

#### Model list, governing equations and boundary conditions

Various computational models have been used to perform flow shear stress and plaque stress and strain calculations, seeking their associations with plaque progression and rupture. Table 2 lists seven models considered in this paper. The assumptions, governing equations and boundary conditions for these models can be found in our previous publication (Wang H et al. 2015; Wang Q et al. 2017; Yang et al. 2009). These models were selected in this paper to compare and investigate the impact of circumferential shrink, axial shrink, FSI and FSI with cyclic bending on model solution behaviors (plaque stress/strain and flow shear stress). The FSI model with cyclic bending (M3) is the best approximation to the vessel physical reality among the seven models and was used as the gold standard when applicable. For this model, blood flow was assumed to be laminar, viscous, incompressible and Newtonian. The incompressible Navier–Stokes equations with arbitrary Lagrangian–Eulerian (ALE) formulation were used as the governing equations. Physiological pressure conditions were prescribed at both inlet and outlet. No-slip conditions and natural traction equilibrium conditions were assumed at all interfaces between fluid and vessel and between plaque components. Putting these together, we have (summation convention is used):

1 $$\rho \left(\partial \mathbf{u}/\partial t+\left(\left(\mathbf{u}-{\mathbf{u}}_{g}\right)\bullet \nabla \right)\mathbf{u}\right)=-\nabla p+\mu {\nabla }^{2}\mathbf{u}$$

2 $$\nabla \bullet \mathbf{u}=0$$

3 $${\mathbf{u}|}_{\Gamma }=\partial \mathbf{x}/\partial t, {\partial \mathbf{u}/\partial n|}_{\mathrm{inlet},\mathrm{outlet}}=0$$

4 $${p|}_{\mathrm{inlet}}={p}_{\mathrm{in}}\left(t\right), {p|}_{\mathrm{outlet}}={p}_{\mathrm{out}}\left(t\right), p{|}_{\mathrm{out boundary}}=0,$$

5 $$\rho {v}_{i,tt}={\sigma }_{ij,j}, i,j=\mathrm{1,2},3, \mathrm{sum over} j,$$

6 $${\varepsilon }_{ij}={v}_{i,j}+{v}_{j,i}+{v}_{\alpha ,i}{v}_{\alpha ,j}/2, i,j,\alpha =\mathrm{1,2},3$$

7 $${{\sigma }_{i,j}\bullet {n}_{j}|}_{{\mathrm{out}}_{\mathrm{wall}}}=0$$

8 $${{\sigma }_{ij}^{r}\bullet {n}_{j}|}_{\mathrm{interface}}={{\sigma }_{ij}^{s}\bullet {n}_{j}|}_{\mathrm{interface}},$$

9 $${\mathbf{x}}_{{{\text{center}}}} = {\mathbf{x}}_{{{\text{bending}}}} \left( t \right),$$

where **u** and *p* are fluid velocity and pressure, **x**_center_ is the vessel centerline, **x**_bending_ is the imposed cyclic bending condition derived from patient angiography movie, **u**_g_ is the mesh velocity, $\mu$ is the dynamic viscosity, $\rho$ is density, $\Gamma$ stands for vessel inner boundary,$f$_**∙**, *j*_ stands for derivative of *f* with respect to the *j*th variable, ${\varvec{\sigma}}$ is the stress tensor (superscripts indicate different materials), ${\varvec{\varepsilon}}$ is the strain tensor, **v** is the solid displacement vector, superscript letters *r* and *s* were used to indicate different materials (fluid or different plaque components). For simplicity, all material densities were set to 1 in this paper.

**Table 2:** List of models used in this paper (“Y”: feature included; “N”: feature not included)

Models	Circumferential shrink process	Axial shrink	Cyclic bending	FSI
M1: 2D with circumferential shrink	Y	N	N	N
M2: 2D without circumferential shrink	N	N	N	N
M3: 3D FSI model with cyclic bending	Y	Y	Y	Y
M4: 3D FSI model without cyclic bending	Y	Y	N	Y
M5: 3D TL structure-only model	Y	Y	N	N
M6: 3D structure-only vessel model	Y	Y	Y	N
M7: 3D fluid-only vessel model	N	N	N	N

#### The anisotropic Mooney–Rivlin model for material properties and parameter values

Coronary vessel material was assumed to be hyperelastic, anisotropic, nearly-incompressible and homogeneous. The anisotropic Mooney–Rivlin model was used to describe the material properties of the vessel tissue (Bathe 2002). The strain energy density function was:

10 $$W = c_{1} \left( {I_{1} {-}3} \right) \, + \, c_{2} \left( {I_{2} {-}3} \right) \, + \, D_{1} \left[ { \, \exp \left( {D_{2} \left( {I_{1} {-}3} \right)} \right) \, {-}1} \right] + \left( {K_{1} /K_{2} } \right) \, \exp \left[ {K_{2} \left( {I_{4} - 1} \right)^{2} - 1} \right],$$

11 $${\text{I}}_{1} = \sum {C_{ii} } ,\;\,{\text{I}}_{2} = {\raise0.5ex\hbox{$\scriptstyle 1$} \kern-0.1em/\kern-0.15em \lower0.25ex\hbox{$\scriptstyle 2$}}[I_{1}^{2} - C_{ij} C_{ij} ],$$

where I_1_ and I_2_ are the first and second invariants of right Cauchy–Green deformation tensor **C** defined as ***C*** = [*C*_*ij*_] = **X**^**T**^**X, X** = [X_ij_] = [∂x_i_/∂a_j_], (x_i_) is current position, (a_i_) is original position, I_4_ = *C*_*ij*_(**n**_*c*_)_*i*_(**n**_*c*_)_*j*_*,* **n**_*c*_ is the unit vector in the circumferential direction of the vessel, c_1_, c_2_, D_1_, D_2_, K_1_ and K_2_ are material parameters. Plaque components were assumed to be isotropic, and the isotropic Mooney–Rivlin material model was used to describe their material properties:

12 $$W = c_{1} \left( {I_{1} {-}3} \right) \, + \, c_{2} \left( {I_{2} {-}3} \right) \, + \, D_{1} \left[ { \, \exp \left( {D_{2} \left( {I_{1} {-}3} \right)} \right) \, {-}1} \right],$$

In this paper, the following parameter values were chosen: vessel tissue/fibrous cap, *c*_1_ = −1312.9 kPa, *c*_2_ = 114.7 kPa, *D*_1_ = 629.7 kPa, *D*_2_ = 2.0, *K*_1_ = 35.9 kPa, *K*_2_ = 23.5; Lipid: *c*_1_ = 0.5 kPa, *c*_2_ = 0 kPa, *D*_1_ = 0.5 kPa, *D*_2_ = 1.5; Calcification: *c*_1_ = 92.0 kPa, *c*_2_ = 0 kPa, *D*_1_ = 36.0 kPa, *D*_2_ = 2.0 (Tang et al. 2004; Kural et al. 2012). Axial shrinkage was set at 10% in our models (if applied) because atherosclerotic vessels are stiffer than healthy vessels. Circumferential pre-shrink process was performed to ensure that the vessel would regain its in vivo geometry when pressure conditions were imposed (Guo et al. 2017). It should be noted that circumferential shrinkage rates were determined by material properties using an iterative process and could not be specified arbitrarily.

### Model simplifications and comparisons

Starting from the full 3D FSI model given in Sect. 2.1, various simplifications were made to get the six simplified models. 3D TL model was motivated by the need to build plaque models with reduced labor cost for possible practical clinical implementations. 3D TL model uses a 2D slice and adds a 0.5 mm thickness so that axial shrink–stretch process could be performed. The difference between 3D TL model and 2D model is that 3D TL model includes axial shrinkage, while 2D model does not. Clearly 3D TL model does not have the full 3D plaque structure and vessel curvature which 3D FSI model and structure-only model do have. 3D FSI model includes circumferential and axial shrinkage, while 3D fluid-only vessel model does not. Other model differences between 2D, 3D TL, 3D structure-only, 3D fluid-only and 3D FSI models are self-evident. Uniform pressure conditions were specified on lumen for all 2D, 3D TL and structure-only models:

13 $${p|}_{\mathrm{lumen}}={p}_{\mathrm{in}}\left(t\right), p{|}_{\mathrm{out boundary}}=0,$$

The modeling process with or without circumferential shrink process was used to quantify the influence of zero-load condition on in vivo image-based 2D models (M1 versus M2). 3D FSI models with or without cyclic bending process were used to study the effect of heart motion for coronary artery (M3 versus M4). The differences between 2D model, 3D TL model and 3D FSI model of different patients were compared to determine a better modeling method (cost-efficient and accurate) for possible practical clinical implementations (M1 and M5 versus M3). It has been long argued that the extra labor cost of 3D FSI models may not be necessary if 3D structure-only or fluid-only models could provide reasonable approximations. Differences between 3D structure-only vessel model and 3D FSI model were compared to explore their differences among different patients (M3 versus M6). With the change of blood pressure, the structure of the vessel wall changes. 3D fluid-only vessel model and 3D FSI model in different patients were compared to explore their differences in flow shear stress (FSS) calculations (M3 versus M7).

### Mesh generation and solution method

A component-fitting mesh-generation process (Yang et al. 2009) was used to generate mesh for our models. The finite element models were solved by a commercial finite element software ADINA (Adina R & D, Watertown, MA, USA) following established procedures (Wang H et al. 2015; Wang Q et al. 2017; Yang et al. 2009). Mesh analysis was performed by refining mesh density by 10% until solutions became mesh independent, i.e., changes of subsequent solutions became less than 1%. Three cardiac cycles were simulated for all models, and the solution in the third period was taken as the final results for analysis since the solutions for the second and third cycles became almost identical.

### Data analysis

Three hundred and eighty-eight (388) slices from seven patients were evaluable for our study. For each slice, flow shear stress (FSS), plaque stress and strain values from 100 evenly-spaced points at the lumen were obtained for analysis. Since stress and strain are tensors, maximum principal stress and strain values at each lumen point were used as the representative scalar values for easy comparison, and denoted as plaque wall stress (PWS) and strain (PWSn) for convenience. The following notations and formulas were used in our calculations:

14 $$\Delta {\text{PWS}}_{i} = \left| {{\text{PWS}}_{p,i} - {\text{PWS}}_{q, i} } \right|$$

15 $${\text{Mean}} \Delta {\text{PWS}} = \mathop \sum \limits_{i = 1}^{n} \Delta {\text{PWS}}_{i} /n$$

16 $${\text{Mean}} {\text{PWS}}_{p} = \mathop \sum \limits_{i = 1}^{n} {\text{PWS}}_{p, i} /n$$

17 $${\text{Relative}} {\text{Error}} = {\text{Mean}} \Delta {\text{PWS}}/{\text{Mean}} {\text{PWS}}_{p}$$

where *i* is the point index of lumen points and ${\mathrm{PWS}}_{p,i}$ and ${\mathrm{PWS}}_{q,i}$ are the PWS values of the *i*th point in Model *p* and Model *q*, respectively. Summation was done at slice, patient, and all patient level as needed for our comparison purpose. “n” is the total points for the summation being performed. PWSn and FSS calculations were done in the same way.

## Results

### Construction time of 2D and 3D model

Table 3 summarizes the time cost of the seven simulation modeling strategies. Currently, the time cost of constructing a 2D model or a 3D TL model for a plaque slice is less than 10 min. The time needed to solve a 2D model or a 3D TL model was less than 2 min. However, it took more than one week for a trained researcher to construct a 3D FSI model. In addition, more than 10 h was required to solve a full 3D transient FSI model (Dell Workstation, Precision 5810). The time cost of constructing a 3D fluid-only vessel model or a 3D structure-only vessel model was more than 2 or 3 days, respectively. Compared with the 3D FSI model, 2D model and 3D TL model can provide timely plaque mechanics analysis for potential clinical application and commercial implementations.

**Table 3:** Time cost of the seven models

Models	Building time	Computing time
M1	< 10 min/slice	< 2 min/slice
M2	< 10 min/slice	< 2 min/slice
M3	> 1 week/vessel	> 10 h/vessel
M4	> 1 week/vessel	> 10 h/vessel
M5	< 10 min/slice	< 2 min/slice
M6	> 3 days/vessel	> 5 h/vessel
M7	> 2 days/vessel	> 10 h/vessel

### 2D models without circumferential shrink process produced higher PWS and PWSn values

**Fig. 2:**
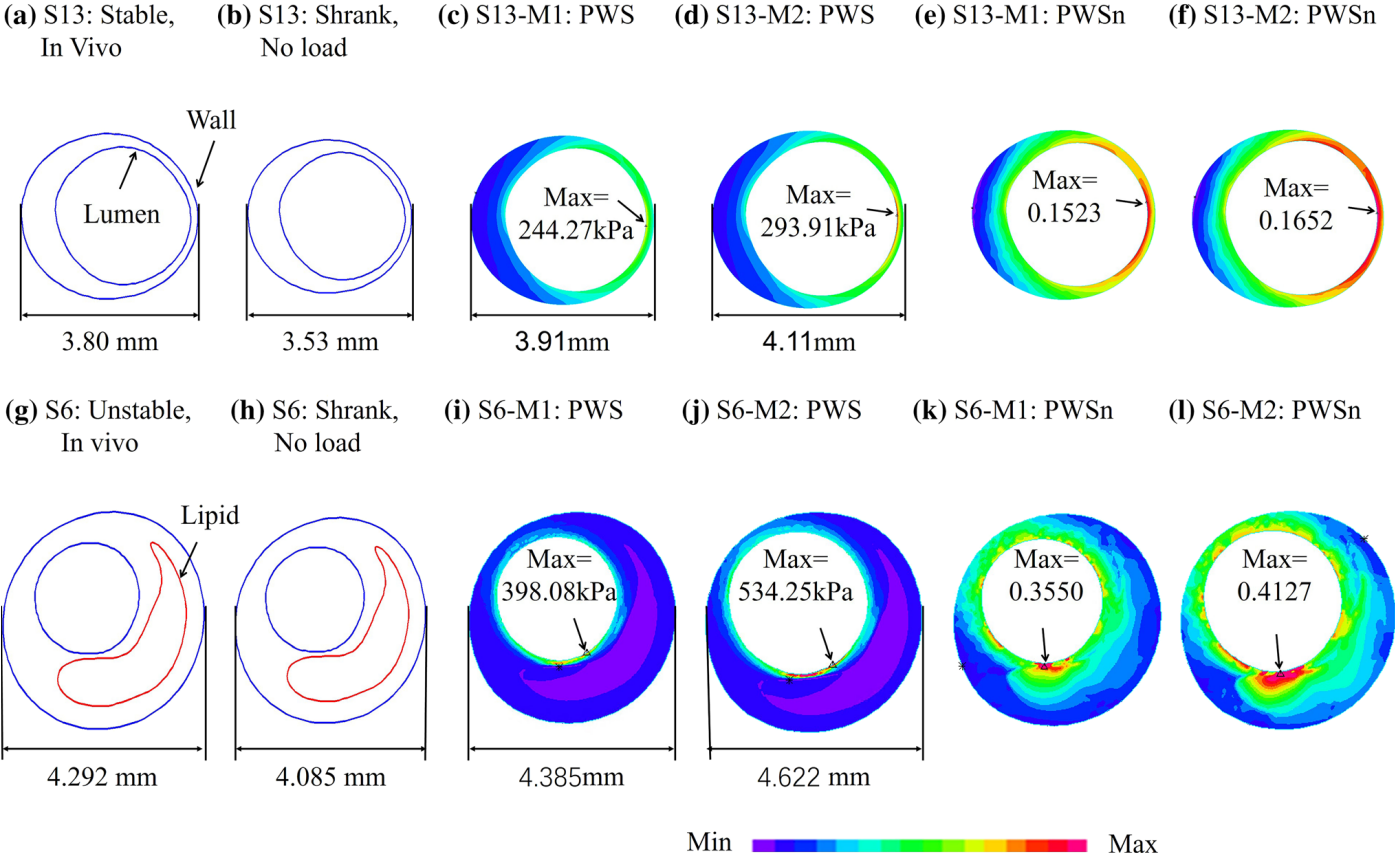
PWS and PWSn plots of M1 and M2 models showing impact of circumferential shrink process on 2D models. M1: 2D with circumferential shrink; M2: 2D without circumferential shrink

To demonstrate model differences with and without circumferential shrinkage, Fig. 2 shows PWS and PWSn plots of one stable plaque (S13 from P1) with no lipid-rich necrotic core (lipid for short) and one unstable plaque (S6 from P5) with a large lipid-rich necrotic core and a thin fibrous cap from two models (M1 and M2). To observe PWS and PWSn patient variations, Table 4 lists PWS and PWSn mean values from M1 and M2 for each patient and the relative errors of M2 using M1 values as the baseline. Mean PWS and PWSn relative errors were 17.26% and 7.13% for the seven patients combined, respectively. PWS relative error patient variations ranged from 9.79% to 29.65%, while PWSn relative error variation ranged from 3.87–13.40%.

**Table 4:** PWS, PWSn and errors for seven patients obtained using M1 and M2 models

Patients	PWS (kPa)			PWSn		
	M1	M2	Error (%)	M1	M2	Error (%)
P1	88.6	98.5	15.40	0.0934	0.0969	5.85
P2	96.0	107.9	14.31	0.0963	0.1001	4.93
P3	97.0	124.0	29.35	0.0860	0.0945	12.46
P4	77.4	85.5	11.13	0.0882	0.0918	4.64
P5	152.5	190.5	**29.65**	0.1929	0.2070	**13.40**
P6	72.3	80.2	11.20	0.0846	0.0882	4.77
P7	95.4	104.0	**9.79**	0.0987	0.1019	**3.87**
Ave	97.0	112.9	17.26	0.1057	0.1115	7.13

M1: 2D with circumferential shrink; M2: 2D without circumferential shrink. Errors were calculated using M1 as the base

The maximum and minimum values of errors from the seven patients were indicated in bold

### Influence of cyclic bending process on coronary artery modeling

**Fig. 3:**
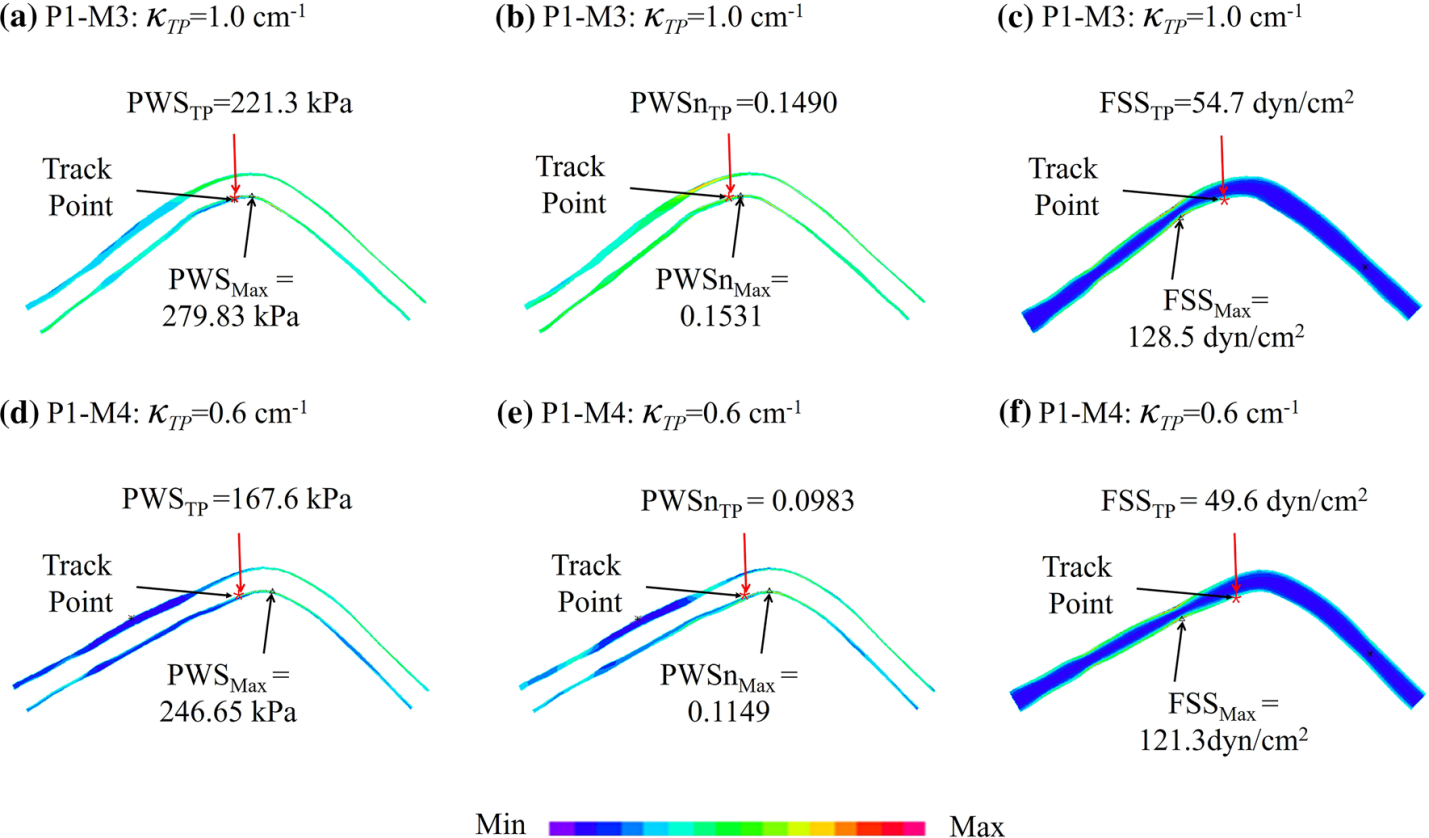
The influence of curvature change of coronary artery with cardiac motion (M3 vs M4) on PWS, PWSn and FSS calculations. M3: 3D FSI model with cyclic bending; M4: 3D FSI model without cyclic bending. TP: tracking point with the greatest curvature change. ${\kappa }_{TP}$: curvature at tracking point

Considering the effect of cardiac motion on coronary arteries, 3D FSI model with cyclic bending (M3) and 3D FSI model without cyclic bending (M4) were constructed. Figure 3 shows the PWS, PWSn and FSS plots from M3 and M4 for P1. Table 5 summarizes PWS and PWSn mean values and maximum FSS (Max-FSS) values from M3 and M4 for each patient. The relative errors of M4 using M3 values as the baseline were given. The average relative error value of PWS in M3 and M4 models of seven patients was 11.50%, and the variation range was 7.16%-14.88%. The average relative error value of PWSn calculated by M3 and M4 models in seven patients was 14.55% (ranged from 9.48% to 19.24%). The average relative error value of Max-FSS in M3 and M4 models of seven patients was 2.59%, and the variation range was 0.46%-6.29%.

**Table 5:** PWS, PWSn, FSS values from M3 and M4 models for seven patients under maximum pressure condition

Patient	PWS (kPa)			PWSn			Max-FSS (dyn/cm^2^)		
	M3	M4	Error (%)	M3	M4	Error (%)	M3	M4	Error (%)
P1	79.2	75.6	12.08	0.0713	0.0628	15.61	136.9	132.7	3.04
P2	82.8	81.0	**7.16**	0.0682	0.0634	10.29	167.4	171.0	2.13
P3	86.4	86.5	12.26	0.0696	0.0708	13.71	36.8	37.1	0.90
P4	71.6	66.0	13.64	0.0721	0.0598	**19.24**	179.4	178.5	**0.46**
P5	113.2	113.4	8.08	0.1535	0.1438	**9.48**	66.3	62.1	**6.29**
P6	57.6	54.5	**14.88**	0.0648	0.0599	15.29	223.5	228.3	2.15
P7	84.4	78.7	12.39	0.0776	0.0647	18.21	276. 3	267.6	3.16
Ave	82.2	79.4	11.50	0.0824	0.0750	14.55	135.1	153.6	2.59

M3: 3D FSI model with cyclic bending; M4: 3D FSI model without cyclic bending. Errors were calculated using M3 as the base

The maximum and minimum values of errors from the seven patients were indicated in bold

The curvature of the coronary arteries at different locations varies with the cyclic bending process. To observe the influence of curvature change of coronary artery on PWS, PWSn and FSS calculation, curvature changes were tracked at all nodal points of the interface of lumen and the inner surface of the vessel wall. Figure 3 shows the tracking point (TP) with greatest curvature change and provided PWS, PWSn and FSS values at that location. Table 6 lists the PWS, PWSn and FSS values from M3 and M4 models for seven patients at the locations with the greatest curvature change and the relative errors of M4 using M3 values as the baseline. Mean PWS, PWSn and FSS relative errors were 30.13%, 23.25% and 6.75% for the seven patients combined, respectively. PWS relative error patient variations ranged between 15.07% and 49.52%, while the PWSn relative error patient variation range was 7.48% -34.05%. FSS relative error patient variation range was more moderate at 1.52% -12.95%.

**Table 6:** PWS, PWSn and FSS values from M3 and M4 models for seven patients at the locations with the greatest curvature change and the relative errors of M4 using M3 values as the baseline

Patient	PWS (kPa)			PWSn			FSS (dyn/cm^2^)		
	M3	M4	Error (%)	M3	M4	Error (%)	M3	M4	Error (%)
P1	221.3	167.6	24.27	0.1490	0.0983	**34.05**	54.7	49.6	9.36
P2	290.5	163.2	43.82	0.1240	0.0947	23.68	41.8	36.4	**12.95**
P3	129.5	103.4	20.13	0.1174	0.1332	13.49	27.6	28.9	5.02
P4	108.1	54.6	**49.52**	0.0770	0.0570	26.00	139.1	126.1	9.38
P5	169.9	195.6	**15.07**	0.1734	0.1864	**7.48**	47.5	46.8	**1.52**
P6	112.4	92.6	17.54	0.1026	0.0734	28.49	59.3	57.0	3.82
P7	262.5	156	40.57	0.1399	0.0986	29.53	123.0	116.6	5.19
Ave	184.9	133.3	30.13	0.1262	0.1059	23.25	70.4	65.9	6.75

The maximum and minimum values of errors from the seven patients were indicated in bold

### Comparison of 2D model, 3D TL model and 3D FSI model

**Fig. 4:**
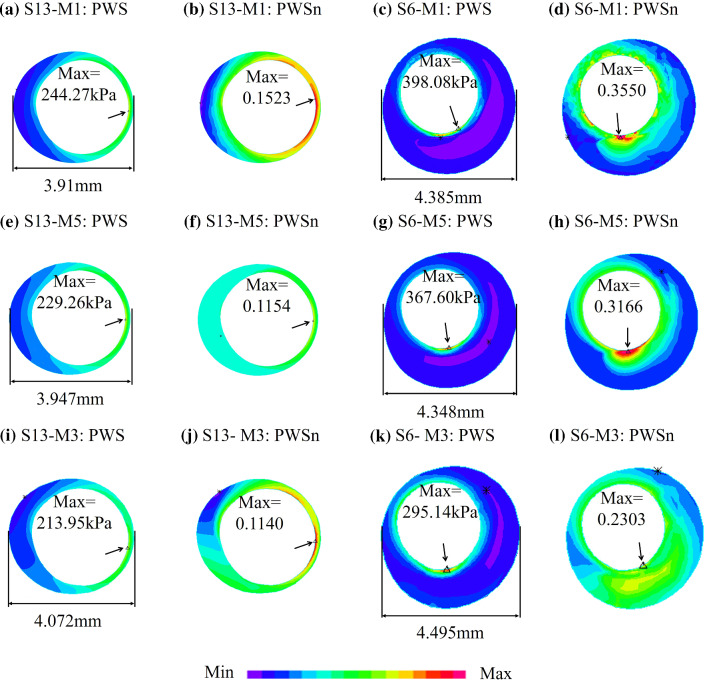
PWS and PWSn plots from M1, M3 and M5 showing PWS and PWSn differences between 2D, 3D TL and 3D FSI models. M1: 2D with circumferential shrink; M3: 3D FSI model with cyclic bending; M5: 3D TL structure-only model

To demonstrate model differences between 2D, 3D TL and 3D FSI models, Fig. 4 provides PWS and PWSn plots of one stable plaque (S13) and one unstable plaque (S6) from three models (M1, M3 and M5). Table 7 lists PWS and PWSn mean values from M1 and M3 for each patient and the relative errors of M1 using M3 values as the baseline. Mean PWS and PWSn relative errors were 33.49% and 34.18% for the seven patients combined, respectively. PWS relative error variation range was 18.72–59.54%, while PWSn relative error variation range was 24.04%-46.07%. Table 8 lists PWS and PWSn mean values from M5 and M3 for each patient and the relative errors of M5 using M3 values as the baseline. Mean PWS and PWSn relative errors were 22.40% and 23.08% for the seven patients combined, respectively. PWS relative error variation range was 14.12–42.24%, while PWSn relative error variation range was 11.80–41.90%. Taking the M3 model as the standard, mean PWS and PWSn relative errors of M5 model of seven patients were 11.09% and 11.1% lower than that of M1 model.

**Table 7:** PWS, PWSn from M1 and M3 for seven patients and errors of M1 were calculated using M3 values as the baseline

Patient	PWS (kPa)			PWSn		
	M1	M3	Error (%)	M1	M3	Error (%)
P1	88.61	79.24	21.33	0.0934	0.0713	32.91
P2	96.00	82.79	20.30	0.0963	0.0682	41.17
P3	97.02	86.35	**59.54**	0.0860	0.0696	**46.07**
P4	77.41	71.55	**18.72**	0.0882	0.0721	**24.04**
P5	152.45	113.22	48.74	0.1929	0.1535	27.94
P6	72.33	57.56	43.81	0.0846	0.0648	38.50
P7	95.42	84.40	21.99	0.0987	0.0776	28.61
Ave	97.03	82.16	33.49	0.1057	0.0824	34.18

M1: 2D with circumferential shrink; M3: 3D FSI model with cyclic bending

The maximum and minimum values of errors from the seven patients were indicated in bold

**Table 8:** PWS, PWSn values from M3 and M5 for seven patients

Patient	PWS (kPa)			PWSn		
	M5	M3	Error (%)	M5	M3	Error (%)
P1	82.40	79.24	**14.12**	0.0640	0.0713	14.58
P2	90.89	82.79	15.03	0.0674	0.0682	**11.08**
P3	82.93	86.35	**42.24**	0.0693	0.0696	35.78
P4	72.75	71.55	16.50	0.0592	0.0721	19.72
P5	96.94	113.22	22.48	0.1756	0.1535	19.17
P6	68.79	57.56	29.86	0.0777	0.0648	**41.90**
P7	82.27	84.40	16.54	0.0636	0.0776	19.30
Ave	82.42	82.16	22.40	0.0824	0.0824	23.08

M3: 3D FSI model with cyclic bending; M5: 3D TL structure-only model. Errors of M5 were calculated using M3 values as the baseline

The maximum and minimum values of errors from the seven patients were indicated in bold

### Comparison of 3D structure-only vessel model and 3D FSI model

**Fig. 5:**
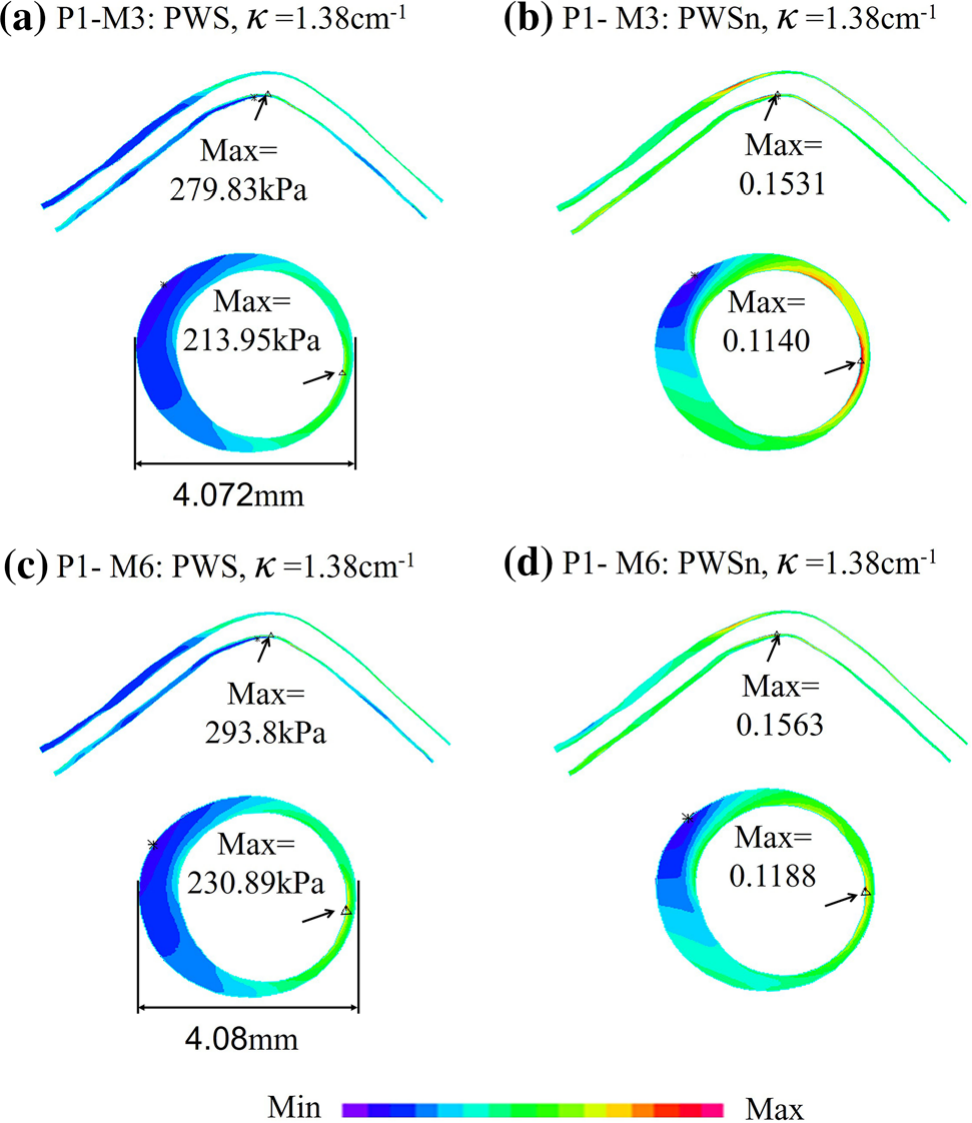
PWS and PWSn plots from M3 and M6 showing computational differences between 3D structure-only model and 3D FSI models. M3: 3D FSI model with cyclic bending; M6: 3D structure-only vessel model

Figure 5 shows the PWS and PWSn plots of P1 from M3 and M6 models to demonstrate model differences between 3D FSI and 3D structure-only models. Table 9 summarizes PWS and PWSn mean values from M3 and M6 for each patient and the relative errors of M6 using M3 values as the baseline. Mean PWS and PWSn relative errors were 4.38% and 1.78% for the seven patients combined, respectively. PWS relative error variation range was 0.29–8.27%, while PWSn relative error variation range was 0.22–3.17%.

**Table 9:** PWS, PWSn values from M3 and M6 for seven patients

Patient	PWS(kPa)			PWSn		
	M3	M6	Error (%)	M3	M6	Error (%)
P1	79.24	83.24	5.04	0.0713	0.0728	2.09
P2	82.79	87.00	5.09	0.0682	0.0697	2.15
P3	86.35	86.61	**0.29**	0.0696	0.0698	**0.22**
P4	71.55	74.34	3.90	0.0721	0.0732	1.54
P5	113.22	115.01	1.61	0.1535	0.1542	0.60
P6	57.56	62.32	**8.27**	0.0648	0.0665	2.70
P7	84.40	89.49	6.46	0.0776	0.0797	**3.17**
Ave	82.16	85.43	4.38	0.0824	0.0837	1.78

M3: 3D FSI model with cyclic bending; M6: 3D structure-only vessel model. Errors of M6 were calculated using M3 values as the baseline

The maximum and minimum values of errors from the seven patients were indicated in bold

### Comparison of 3D fluid-only vessel model and 3D FSI model

**Fig. 6:**
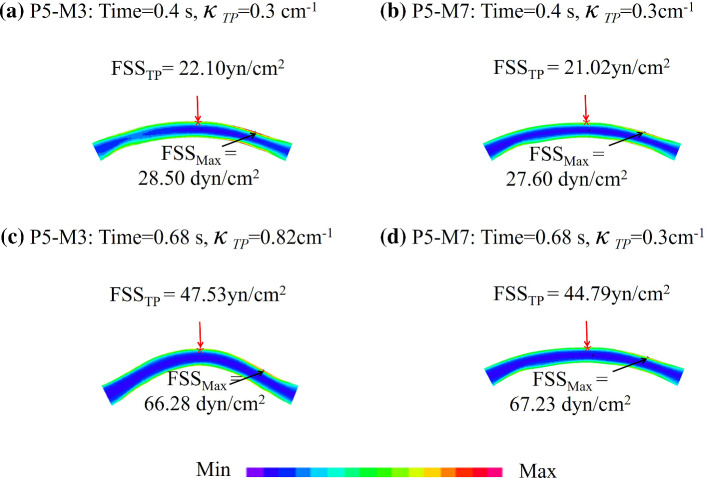
FSS plots under maximum and minimum pressure conditions changed from M3 and M7 show computational differences between 3D FSI model and 3D fluid-only models. M3: 3D FSI model with cyclic bending; M7: 3D fluid-only vessel model. TP: tracking point with the greatest curvature change. ${\kappa }_{TP}$: curvature at tracking point

Figure 6 shows the FSS plots under maximum and minimum pressure conditions from M3 and M7 show computational differences between M3 and M7 models of P5. Table 10 summarizes the maximum FSS (Max-FSS), minimum FSS (Min-FSS) and average FSS (Ave-FSS) from M3 and M7 for each patient under maximum and minimum pressure conditions (P_max_ and P_min_) and the averages over time. The relative errors of M7 using M3 values as the baseline were given. Mean Max-FSS, Min-FSS and Ave-FSS relative errors in M3 and M7 models under P_max_ were 4.02%, 11.29% and 5.45% for the 7 patients combined, respectively. Max-FSS relative error patient variations ranged between 0.56% and 9.52%, while Min-FSS relative error patient variation range was 3.59%-36.63%. Ave-FSS relative error patient variation range was more moderate at 4.87–6.25%. Mean Max-FSS, Min-FSS and Ave-FSS relative errors in M3 and M7 models under P_min_ were 3.09%, 10.17% and 1.85% for the seven patients combined, respectively. Max-FSS relative error patient variations ranged between 0.19% and 7.54%, while Min-FSS relative error patient variation range was 2.83–23.80%. Ave-FSS relative error patient variation range was more moderate at 0.38–2.85%. Mean Max-FSS, Min-FSS and Ave-FSS relative errors in M3 and M7 models under the averages over time were 3.44%, 9.61% and 2.88% for the seven patients combined, respectively. Max-FSS relative error patient variations ranged between 0.62%-7.94%, while Min-FSS relative error patient variation range was 1.13–30.15%. Ave-FSS relative error patient variation range was more moderate at 2.33–3.29%.

**Table 10:** FSS values from M3 and M7 for seven patients

Patient	Max-FSS (dyn/cm^2^)			Min-FSS (dyn/cm^2^)			Ave-FSS (dyn/cm^2^)		
	M3	M7	Error (%)	M3	M7	Error (%)	M3	M7	Error (%)
*Under maximum pressure condition*									
P1	136.9	141.0	3.03	14.9	15.8	6.13	54.4	57.3	5.27
P2	167.4	172.2	2.83	12.6	14.3	13.93	51.6	53.3	**4.87**
P3	36.8	39.8	8.41	6.4	6.0	5.66	19.1	19.7	5.38
P4	179.4	183.5	2.33	21.1	22.6	7.03	66.9	70.9	6.06
P5	66.3	67.23	1.43	12.2	16.7	**36.63**	35.0	36.8	5.03
P6	223.5	244.8	**9.52**	18.4	19.0	**3.59**	77.5	79.6	5.29
P7	276.3	277.9	**0.56**	15.2	16.1	6.05	94.4	97.8	**6.25**
Ave	135.1	160.9	4.02	14.4	15.8	11.29	57.0	59.3	5.45
*Under minimum pressure condition*									
P1	43.7	44.7	2.21	6.1	6.4	5.45	18.8	19.0	1.11
P2	55.0	55.1	**0.19**	5.2	5.8	11.93	17.4	17.8	2.26
P3	19.4	20.8	**7.54**	4.2	3.8	9.75	10.7	10.9	2.04
P4	35.9	36.6	1.96	5.8	6.2	6.70	13.8	14.1	2.31
P5	28.5	27.6	3.20	7.0	8.7	**23.80**	17.2	17.2	**0.38**
P6	47.8	49.2	2.86	5.5	5.4	**2.83**	16.1	16.5	1.97
P7	95.9	99.4	3.68	8.9	7.9	10.74	35.5	36.5	**2.85**
Ave	46.6	47.6	3.09	6.1	6.3	10.17	18.5	18.9	1.85
*Average over cardiac cycle*									
P1	108.5	111.5	2.76	12.9	13.6	5.73	44.2	45.3	2.51
P2	134.9	136.9	1.48	10.8	12.0	11.11	41.6	42.8	3.06
P3	28.4	30.6	**7.94**	5.4	5.0	7.41	15.1	15.5	**2.33**
P4	120.8	123.3	2.10	15.4	16.4	6.78	45.3	46.7	3.15
P5	45.0	45.3	**0.62**	10.1	13.1	**30.15**	26.8	27.7	**3.29**
P6	175.0	188.2	7.58	15.3	16.0	4.96	59.8	61.3	2.57
P7	231.5	235.2	1.61	13.9	13.8	**1.13**	80.6	83.2	3.24
Ave	120.6	124.4	3.44	12.0	12.8	9.61	44.8	46.1	2.88

M3: 3D FSI model with cyclic bending; M7: 3D fluid-only vessel model. Errors of M7 were calculated using M3 values as the baseline

The maximum and minimum values of errors from the seven patients were indicated in bold

## Discussion

### The importance of studying multiple patients

It is well accepted that different models may provide different computational results. It is also well known that patient-specific modeling and mechanical information are important for diagnosis and precision medicine where medication and treatment strategies would be decided on a patient-by-patient basis. Previous model comparison studies often use single-patient data or idealized geometries and their results normally report “Model A over-estimates PWS by 30% compared with Model B (the gold standard).” Holzapfel et al. (2002) introduced a layer-specific 3D anisotropic model (baseline model, or the gold standard) based on in vitro magnetic resonance imaging of a human stenotic postmortem artery. Model differences between the baseline model and other three simplified models (model without axial pre-stretch, model with plane strain and isotropic model) went as high as 600%. Yang et al. (2009) compared maximum of Stress-P_1_ (maximum principal stress) on a cut surface of from five different models using one patient data. Compared to the isotropic model (Model 1, no bending, no axial stretch), maximum Stress-P_1_ values on the cut surface with maximum bending (where applicable) from Model 2 (anisotropic, no bending, no stretch), Model 3 (anisotropic, with bending, no stretch) and Model 4 (anisotropic with bending and stretch) were 63%, 126% and 345% higher than that from Model 1, respectively. Guo et al. used in vivo vessel material properties in their coronary plaque models and they reported that average cap strain values using in vivo material models were 150%-180% higher than those from the ex vivo material models. The corresponding percentages for average cap stress values were 50–75% (Guo et al. 2017). Wang L. et al. used a near-idealized plaque geometry to study the impact of residual stress (opening angle), axial shrink–stretch and circumferential pre-shrink on plaque stress/strain calculations. They reported that the model with axial stretch, circumferential shrink, but omitting opening angle overestimated lumen and cap stress by 182% and 448%, respectively (Wang L. et al. 2017). While the large percentage values demonstrated the importance of using the “right” models for plaque mechanical analysis, it is natural to move on to multi-patient studies to investigate patient variations in model differences. Table 11 lists model comparison difference ranges reported in this paper. For seven patients, PWS difference from 2D models with/without circumferential shrink process varied from 9.79 to 29.65%. PWS change at location with greatest curvature change from 3D FSI models with/without cyclic bending varied from 15.07 to 49.52%. As for hydrodynamic calculation difference analysis, the Min-FSS difference between 3D fluid-only vessel models and 3D FSI models varied from 3.59 to 36.63% for seven patients. Patient variations for other model comparisons gave similar results. Caution should be taken when interpreting computational results from different models, with patient variations kept in mind.

**Table 11:** Summary of model comparison patient variation ranges

Models compared	PWS (%)	PWSn (%)	Max-FSS (%)	Min-FSS (%)	Ave-FSS (%)
2D models with/without circumferential shrink (M1 vs. M2)	9.79–29.65	3.87–13.40	N/A	N/A	N/A
3D FSI models with/without cyclic bending (M3 vs. M4)	7.16–14.88	9.48–19.24	0.46–6.29	N/A	N/A
3D FSI models with/without cyclic bending at locations with max curvature change (M3 vs. M4)	15.07–49.52	7.48–34.05	1.52–12.95	N/A	N/A
2D model vs. 3D TL model (M1 vs. M3)	18.72–59.54	24.04–46.07	N/A	N/A	N/A
3D TL model vs. 3D FSI model (M3 vs. M5)	14.12–42.24	11.08–41.90	N/A	N/A	N/A
3D structure-only model versus 3D FSI model (M3 vs. M6)	0.29–8.27	0.22–3.17	N/A	N/A	N/A
3D fluid-only model versus 3D FSI model (M3 vs. M7), maximum pressure	N/A	N/A	0.56–9.52	3.59–36.63	4.87–6.25
3D fluid-only model versus 3D FSI model (M3 vs. M7), minimum pressure condition	N/A	N/A	0.19–7.54	2.83–23.80	0.38–2.85
3D fluid-only model versus 3D FSI model (M3 vs. M7), Average over a cardiac cycle	N/A	N/A	0.62–7.94	1.13–30.15	2.33–3.29

Numbers given below are percentages of model solution differences unless otherwise indicated. N/A: not applicable

### The importance of pre-shrink process for image-based models

As shown in Table 4, PWS and PWSn values of the plaques would be overestimated under the models without shrink process. The influence of 2D model without shrink process on PWS calculation is greater than on PWSn calculations. The errors of PWS and PWSn were 17.26% and 7.13%, respectively. Patient variations of PWS errors for the seven patients ranged from 9.79 to 29.65%. This demonstrates that pre-shrinking process and correct initial zero-load geometry are important for models based on in vivo images to obtain accurate computational stress/strain results.

### The influence of cyclic bending on the results of coronary artery simulation modeling

The cyclic bending process has great influence on 3D FSI model calculations, especially at locations with large curvature changes. PWS and PWSn values from 3D FSI models with and without cyclic bending for seven patients showed significant differences at the locations with the greatest curvature change (mean PWS and PWSn relative errors were 30.13% and 23.25%, respectively). There were large patient variations in those errors (PWS: 15.07%–49.52%; PWSn: 7.48–34.05%). Clearly, the effect of heart movement on the coronary plaque model needs to be considered. Cyclic bending is only one step in fully coupling heart motion to plaque models. Fully coupled ventricle-vessel FSI models should be considered in the future.

### 3D TL model was superior to the 2D model

A major limitation of 3D FSI models is the model construction time cost. Compared with 3D FSI models, 2D and 3D TL models have much lower time cost (less than 10 min for each slice). Considering their simplicity and low time consumption, 2D and 3D TL models are more suitable for clinical simulation modeling than 3D FSI models. It is shown in Tables 7 and 8 that 3D TL models provided better approximations to 3D FSI models than 2D models. Considering the time cost and accuracy of the calculations, 3D TL modeling may be a better choice for clinical implementations.

### 3D structure-only models provided good approximation for FSI models

While 3D FSI models may be more realistic, they take much more time to construct. Compared with 3D FSI models (used as the base for comparison), 3D structure-only models had modest errors (4.38% and 1.78% for PWS and PWSn calculation). Structure-only models could provide PWS/PWSn calculations as good approximations to FSI models to save time. For flow simulation, 3D fluid-only models had larger error on Min-FSS calculation (11.29% under maximum pressure condition). And the multi-patient study also showed significant differences among seven patients.

(3.59–36.63%). FSI and flow-only model differences were greater for minimum FSS predictions, which is notable since low FSS is known to be related to plaque progression.

### Model comparisons and other factors affecting plaque mechanical conditions

In addition to model assumptions, plaque morphology (especially cap thickness), component material properties and blood pressure all have large impact on model stress/strain calculations. Figures 2 and 4 provide samples showing model solution differences were noticeably greater for plaques with thin cap and large lipid core. The current paper focused on model comparison and patient variations. Other comparisons using multi-patient data will be our future effort.

### Modeling limitations

Patient-specific and tissue-specific material properties were not used in our study due to the difficulty in obtaining in vivo material data. Zero-stress conditions (opening angle) and multilayer morphology of vessels are also difficult to measure noninvasively in vivo. The resolution of IVUS images is still insufficient to accurately determine thin fibrous cap thickness, and higher resolution imaging approaches such as optical coherence tomography (OCT) should be adopted in the future to improve image segmentation accuracy.

## Conclusions

Model differences had noticeable patient variations that must be considered when interpreting computational results from different models for different patients. Results from seven patients presented in this study showed that PWS and PWSn values would be overestimated by 2D models without shrink process. Cyclic bending process has an influence on the calculation of 3D FSI model, especially in locations with large curvature change. The effect of heart movement on coronary models should be considered. Considering time cost and accuracy of the stress and strain calculations, 3D TL models may be used for the mechanical analysis of atherosclerotic plaques and may be most practical for clinical implementations. FSI and flow-only model differences were greater for minimum FSS predictions, notable since low FSS is related to plaque progression. Structure-only models may provide PWS/PWSn calculations as reasonable approximations to FSI models to save time.
